# Perceived Facilitators and Barriers to Engaging with a Digital Intervention among Those with Food Insecurity, Binge Eating, and Obesity

**DOI:** 10.3390/nu13072458

**Published:** 2021-07-19

**Authors:** Anu Venkatesh, Angela Chang, Emilie A. Green, Tianna Randall, Raquel Gallagher, Jennifer E. Wildes, Andrea K. Graham

**Affiliations:** 1 Department of Medical Social Sciences, Northwestern University Feinberg School of Medicine, Chicago, IL 60611, USA; alankrita.venkatesh@northwestern.edu (A.V.); angela.chang@northwestern.edu (A.C.); 2 Department of Psychology, Rosalind Franklin University for Medical Sciences, Chicago, IL 60064, USA; emilie.green@my.rfums.org; 3 Weinberg College of Arts and Sciences, Northwestern University, Evanston, IL 60208, USA; tiannarandall2024@u.northwestern.edu (T.R.); raquelgallagher2023@u.northwestern.edu (R.G.); 4 Department of Psychiatry & Behavioral Neuroscience, University of Chicago, Chicago, IL 60611, USA; jwildes1@yoda.bsd.uchicago.edu

**Keywords:** food insecurity, binge eating, obesity, user-centered design, digital intervention

## Abstract

Interventions that address binge eating and food insecurity are needed. Engaging people with lived experience to understand their needs and preferences could yield important design considerations for such interventions. In this study, people with food insecurity, recurrent binge eating, and obesity completed an interview-based needs assessment to learn facilitators and barriers that they perceive would impact their engagement with a digital intervention for managing binge eating and weight. Twenty adults completed semi-structured interviews. Responses were analyzed using thematic analysis. Three themes emerged. Participants shared considerations that impact their ability to access the intervention (e.g., cost of intervention, cost of technology, accessibility across devices), ability to complete intervention recommendations (e.g., affordable healthy meals, education to help stretch groceries, food vouchers, rides to grocery stores, personalized to budget), and preferred intervention features for education, self-monitoring, personalization, support, and motivation/rewards. Engaging people with lived experiences via user-centered design methods revealed important design considerations for a digital intervention to meet this population’s needs. Future research is needed to test whether a digital intervention that incorporates these recommendations is engaging and effective for people with binge eating and food insecurity. Findings may have relevance to designing digital interventions for other health problems as well.

## 1. Introduction

Food insecurity, defined as inadequate/inconsistent access to food required for a healthy life [1], is associated with binge eating [2] and binge eating disorder [3]. Binge eating is an eating disorder behavior characterized by eating an unusually large amount of food while experiencing a sense of loss of control [4]. Among U.S. adults, binge eating disorder has an estimated lifetime prevalence of 0.85% [5], which equates to roughly 2.2 million people [6], with higher rates among women than men, and is associated with serious physical and mental health problems including obesity, diabetes, hypertension, sleep problems, as well as mood, anxiety, and substance use disorders [5,7,8].

In a recent review, Hazzard and colleagues [9] conclude that the growing, consistent evidence documenting the cross-sectional relationship between binge eating and food insecurity highlights a need to identify effective ways to intervene on both binge eating and food insecurity, as no interventions have been tested. The mechanism by which food insecurity leads to binge eating is not yet understood, and the authors of the review state that engaging those with lived experiences through qualitative approaches could offer great value toward this goal [9].

We conducted an interview-based needs assessment to learn facilitators and barriers that people with food insecurity, recurrent binge eating, and obesity perceive would impact their engagement with a digital intervention for managing binge eating and weight. To our knowledge, this is the first study to use qualitative approaches to understand the experiences of this population. This work is part of a larger research program to design a digital intervention for people with recurrent binge eating and comorbid obesity, in which we are applying user-centered design methods to engage deeply with intervention end-users to ensure its design meets users’ needs, goals, and preferences [10,11,12,13]. Learning from end-users the factors that would make them more or less likely to engage with a digital intervention is an essential step in designing an intervention that is acceptable, accessible, and effective for those with food insecurity and binge eating.

## 2. Materials and Methods

Participants were recruited from across the U.S. using ResearchMatch [14], an online research recruitment program supported by the National Institutes of Health and created by academic institutions for researchers to advertise studies to >150,000 volunteers; currently, >10,000 researchers across >180 institutions are recruiting on ResearchMatch. Interested individuals completed an online screener. Eligible individuals were non-pregnant English-speaking adults (≥18 years old) who reported ≥12 binge eating episodes in the past 3 months (to signify recurrent binge eating) plus feelings of distress about these episodes, scored ≥2 on the 6-item Short Form Food Security Survey Screener [15], and had a body mass index ≥30 kg/m^2^ based on self-reported height and weight. To reflect intended users of a digital intervention for binge eating and weight management, eligible individuals also had to be interested in losing weight and reducing binge eating and own a smartphone.

This study was approved by the Northwestern University Institutional Review Board. All participants provided informed consent. 

Participants completed a 45-min semi-structured interview, audio-recorded on Zoom, that assessed the relationship between binge eating and food insecurity. One question set asked participants to describe facilitators and barriers to engaging with a digital intervention. Specific prompts included: “If you could have access to an online program that could help you reduce your binge eating and lose weight, what would make it likely for you to do this program?”, “What would get in the way of you doing such a program?”, and “What types of things would be important to you for this program to have?” Following study procedures, participants received a $20 electronic gift card.

Zoom transcribed each audio-recorded interview. Transcripts were then checked for accuracy and deidentified by approved study team members. Two study team members together analyzed all transcripts via thematic analysis [16], which entailed reading the transcripts, generating codes with open inductive coding, applying codes to transcripts, and compiling codes into themes. 

## 3. Results

Twenty participants were interviewed and asked the question set relevant to this study. Participants’ mean age was 46 ± 12.2 (range = 26–69) years old. The sample was 70% (*n* = 14) female, 20% (*n* = 4) male, 5% (*n* = 1) gender nonbinary, and 5% (*n* = 1) did not specify. Regarding race, 35% (*n* = 7) identified as Black/African American, 60% (*n* = 12) White, and 5% (*n* = 1) other; 20% identified as Hispanic/Latino (*n* = 4). Mean BMI was 39.3 ± 6.2 kg/m^2^. Two-thirds (65%; *n* = 13) reported low food security status and 35% (*n* = 7) reported very low food security status. 

Participants expressed different considerations that could impact their engagement with an intervention; these were grouped into three themes, described below. Table 1 presents specific codes and representative quotes.

### 3.1. Ability to Access the Intervention

One theme focused on the ability to access the intervention. Participants voiced concerns about how much an intervention would cost. Past efforts to seek treatment with an eating disorder specialist were unattainable due to the high cost per session. Therefore, participants indicated that an intervention should be free or use a sliding scale or one-fee payment structure. 

Participants said that an accessible intervention also needs to account for the cost of the technology needed to use the program. For example, participants were concerned about whether they could access the program only on certain devices, noting that they could not afford to buy a new device to use the program. As such, participants recommended that the intervention be accessible across multiple device types, like a computer and smartphone, since access to different devices varied by setting (e.g., at work versus home).

### 3.2. Ability to Complete Intervention Recommendations

Another theme focused on the ability to complete recommendations that may be prescribed by an intervention. Participants discussed that an eating-focused intervention would need to consider the affordability of healthy meals. They described how healthy recipes were often financially unrealistic due to the expense of ingredients, and that it may be hard to justify purchasing ingredients if the meals only last one week. Therefore, participants desired meal recommendations and recipes comprised of affordable ingredients that could be repurposed across meals. One participant noted that the intervention could teach people how to “stretch” foods so ingredients and recipes would last for a longer time. Relatedly, participants recommended providing coupons or vouchers for healthy foods, sending food to them, or recommending foods that are accessible with food stamps. 

Additionally, participants shared that their ability to follow through on recommendations for healthy eating could be impeded by their ability to access grocery stores that sell low or reasonably-priced items (e.g., low-cost ingredients for healthy recipes or fresh produce). One participant shared that he did not have the means (i.e., no car or public transportation) to access these types of grocery stores, and only could access the local, higher-priced store. Thus, an intervention could help with obtaining food such as by covering the cost of transportation to the grocery store or facilitating home delivery. Similarly, some participants assumed an intervention would include in-person sessions with various clinicians and indicated that they would want the intervention to include rides to and from appointments with their physician or a dietitian.

Finally, participants wanted an intervention to be personalized to their budget, with recommendations for engaging in healthy behaviors that are tailored to their financial needs.

### 3.3. Preferences for Intervention Features

The third theme focused on preferences for intervention features that would make engagement more likely. 

Participants wanted a program to provide education on the importance of a healthy lifestyle and explain how the intervention would facilitate that goal. They suggested an intervention provide education on nutrition and binge eating, ideas for healthy recipes, and strategies to avoid unplanned/binge eating when they feel the urge to eat. They wanted healthy food ideas presented in a positive way. One participant indicated that they did not want a “diet” that restricts what foods they can and cannot eat. Participants wanted an intervention to represent all body types, address the psychological aspects of weight gain and binge eating (e.g., why their mood may change following a binge), and help them improve their body image. They also wanted clear instructions on how to use the program. One person said the ability to access the program on a computer would be a “plus” given the larger screen size. 

Participants wanted an intervention to include self-monitoring; they would be interested in tracking weight, eating/food, calories, mood, and progress towards goals. One participant described wanting a food journal to monitor eating-related cognitions, but did not want to count calories or “points” based on food intake given negative perceptions of these latter monitoring systems. Another participant suggested incorporating “before and after pictures” to visualize their actual or potential progress. 

Another important feature was personalization. Participants wanted a program to be tailored to their lifestyle, accounting for their personal budgets, time constraints, dietary preferences (e.g., vegetarian/vegan, preferring familiar foods/ingredients), knowledge of foods, and stage in life (e.g., age). They wanted tailored strategies for reducing binge eating and for the intervention to be responsive to changes in their mood. They recommended the program be flexible in time requirements for use (e.g., when, how often) and implementing the strategies (e.g., not requiring a lot of time for food preparation) due to busy schedules and attention span. 

Personalized support from humans (which could include group sessions) or artificial intelligence conversational agents also were noted. Such support could provide encouragement (e.g., offer comfort, help them persevere through frustrations of not seeing quick results), structure, and accountability that is personalized to their stage of treatment/recovery. Participants shared that accountability would be helpful if it was sustained over time versus only offered at the beginning of the program. 

Finally, participants felt the intervention should sustain their motivation and build their self-efficacy by helping them change negative beliefs regarding their capability to achieve goals. They said a reward system would encourage engagement, most commonly in the form of financial rewards. In fact, one participant felt that only financial rewards, versus other rewards like earning points, would motivate behavior change. 

## 4. Discussion

Interventions that address binge eating and food insecurity are needed [9]. This study engaged people with lived experiences to solicit design considerations for a digital intervention for people with recurrent binge eating, obesity, and food insecurity. Informative recommendations emerged. 

Participants described several considerations regarding digital intervention accessibility, namely, costs of the intervention and technology and accessibility across devices. Although delivering a digital intervention may appear feasible given that 85% of U.S. adults own smartphones [17], and everyone in our sample owned a smartphone to represent intended users of a digital intervention, intervention developers and designers need to be attentive to nuances that can affect access. Participants also offered recommendations to support implementing changes for eating and weight management. Given that individuals with food insecurity have inadequate/inconsistent access to food [1], interventions need to consider ways to overcome structural barriers to engaging in healthy behaviors while also improving food insecurity. Participants even described considerations beyond only a digital intervention in terms of facilitating access to physicians and dietitians, highlighting the importance of intervening across multiple levels to facilitate comprehensive lifestyle management. Because few studies have measured the impact of food insecurity interventions on both food insecurity and health outcomes [18], and none have focused on binge eating, work is needed to design interventions that facilitate healthy behaviors and to study their impact on food insecurity and health outcomes, including binge eating. 

Participants also described their preferences for intervention features. While many suggestions like education and self-monitoring are common evidence-based elements of apps for eating disorders [19], these results offer informative specifications for how such components are preferred to be packaged in a digital intervention for this population. For example, results suggest the importance of supporting people in preparing affordable meals (e.g., that stretch beyond a single meal) and that account for the time and expense of obtaining foods. Participants also wanted personalized support from humans or artificial intelligence, with intervention recommendations tailored to factors such as their budget, mood, and schedule—thereby suggesting the intervention leverage advances in technology to increase customization. Designs that pay explicit attention to these considerations will be important for ensuring that the intervention meets this population’s needs. To that end, the next step in our program of research is ensuring that the mobile intervention currently being designed for binge eating and weight management is appropriate to the needs of individuals with comorbid food insecurity. However, any design recommendations would need to be tested in practice, such as through future stages of the user-centered design process [11].

The strength of this study was applying user-centered design methods through a needs assessment to capture qualitative data from target users of a digital intervention who have lived experiences. These methods, and many findings here, have relevance for designing digital interventions for various health problems. Furthermore, participants were recruited nationwide to capture a broad perspective of people with food insecurity. However, the small sample size, and that participants were primarily female, may hinder the generalizability of the results to all people struggling with these issues and precludes subgroup analyses among different ages, races, ethnicities, and genders. Recruitment through ResearchMatch also may not be generalizable to the main population due to characteristics that led them to participate in such a service. Additionally, the “open” nature of the interviews may have helped the research team obtain specific answers from participants.

In summary, people with recurrent binge eating, obesity, and food insecurity offered several design considerations that could facilitate engagement with an intervention that addresses these health problems, and could have value beyond addressing binge eating by ameliorating other health problems for which healthy eating is an important factor. Future studies should aim to incorporate these recommendations into a digital intervention and test its efficacy. 

## Figures and Tables

**Table 1:** Considerations for a digital intervention by people with food insecurity, binge eating, and obesity.

Theme	Representative Quote (s)
Ability to Access the Intervention	
Cost of the intervention	“If it was free, I would do it, yeah. If it costs anything, no I’m not doing it (…) It’s like, I could actually pay for it and just spend less money on food.” (Pt 226) “If you’re very low income, hey, you could maybe get like a free session or maybe will charge you $50 instead of (…) $200 plus dollar range.” (Pt 11)
Cost of the technology	“If my computer died, that would get in the way. I would need to buy a new computer (…) I have 2 credit cards that I have to pay down and they’re each over $1,000, so I can’t really afford to buy a computer program right now.” (Pt 113)
Accessibility on multiple devices	“If I can’t access it on my cell phone, because we can’t access that thing on our computer. (…) I spend most of my time at work (…) something that will restrict my ability to access it would really hurt.” (Pt 77)
Ability to Complete Intervention Recommendations	
Affordable, healthy meals	“I kind of wish (…) they send you good recipes that are healthy, or even, you know, healthy sweets, you know, that doesn’t kill your fat cells, you know. And then, but something that’s not super expensive and hard to bake.” (Pt 204)
Education to help stretch groceries	“Learning how to stretch your food (…) Making it work for more than the two weeks instead of one week.” (Pt 215)
Program-provided coupons, vouchers, or food	“It would be great if those types of programs will come with coupons on the things that, you know, that is healthy for you to eat, or vouchers.” (Pt 246)
Food stamp-accessible	“If it was food stamp accessible, that would work. If not financially, I wouldn’t be able to afford it.” (Pt 244)
Rides to affordable grocery store	“Where I live, I don’t have a car, and there’s no public transportation. (…) If it would cover a ride for me to get to a store that I could afford to buy things, then yeah that would be tremendously helpful. Because if I went to the local store, I wouldn’t be able to do anything.” (Pt 244)
Rides to the physician/dietitian	“It would have to, if possible like my insurance will, pay for rides to the doctors, they would pay for rides to a nutritionist, and so forth like that.” (Pt 244)
Personalized to budget	“(Program that) fits my lifestyle, my budget, my schedule. I don’t think there’s a one size fits all.” (Pt 70)“No, I can’t afford that (seeing a therapist)*,* no. I’d love to, but I can’t afford it now, so, no.” (Pt 162)
Preferences for Intervention Features	
Education	“Tips on healthy eating (…) like when you think you want something to eat, instead of the hand to mouth thing. What is different that we could do at that moment like exercise or, or write a story (…). What can I put it in place of that?” (Pt 189) “Something that can give me healthy food ideas in a positive way.” (Pt 228) “The history that’s known about binge eating, you know; any prior studies might have been done. I love watching and being informed on, you know, any facts that are available.” (Pt 251)
Psychological aspects	“If I go to the doctor and they (say), you’re obese, you need to lose weight. Okay. Well, great. I know that from a physical, but unless you address everything else, you know, again, the mental aspect of it and self-image and all of that, I don’t think that it can be successful.” (Pt 70) “Yeah, I need to change my whole mindset (to adopt a healthy lifestyle).” (Pt 216)
Representation	“I don’t want to see one body type represented. I want to see all body types.” (Pt 246)
Ease of use	“Frankly, just having the information might help (…) you know how the program works and how I would access it. Real basic stuff.” (Pt 230) “If I did the program on a computer, that would be a plus. Um. Because, you know, the computer screen is bigger than a cellphone screen. And I could do it at my leisure. (Pt 113)
Tracking	“I think a tracker would help (…) almost like a food journal aspect to it. I don’t necessarily like the idea of like a calorie counter or like a point system (…), but maybe more of like a cognitive (tracker)” (Pt 226) “Before and after pictures. You know, visual, you know, where you see your progress. Your progress. You want to see good things happen. And even if it’s not progress, a way to show how you can progress. Yeah, try to reach your goal.” (Pt 250)
Personalization	“If there was a program that was going to address everything as far as like, you know, education and to work with me about specific food.” (Pt 70) “Age-appropriate support groups, not just to a large support group and all of everybody but specific ages, you know, that would help with people going through the same problems that you have that are your age.” (Pt 250)
Time requirements	“Something not too long. Something quick you could read real fast, and then go back to whatever you were doing.” (Pt 228) “I don’t go to the store until I absolutely have to. And I try and make just like one trip to get groceries. And so it would have to be something that would work, you know, with that. I don’t, you know, I don’t have time to spend a lot of time preparing food and thinking about it.” (Pt 70)
Support	“Having a check in with a human being would be, or a highly intuitive AI that could learn moods and stuff like that, is an ideal sort of situation.” (Pt 195) “I would like to see some type of like group therapy type sessions. Um, along with some work, things that we can do at home. That would be good.” (Pt 61)
Accountability	“If I was accountable every day. Accountability (from) maybe my family or, you know, someone that doesn’t know me.” (Pt 217)
Motivation	“I probably would not be motivated, I would not believe in myself. I wouldn’t believe that I would be capable of reaching my goals.” (Pt 255) “A reward system that brings someone closer to getting a gift card at the end, if they do really well.” (Pt 113)

## Data Availability

Data are available upon reasonable request from the corresponding author.
